# Complete genome sequence and genetic features of a novel *Pseudomonas* sp. isolate (CAM1A) from tsetse fly gut captured in Dodeo, Cameroon

**DOI:** 10.1186/s12863-025-01398-z

**Published:** 2025-12-13

**Authors:** Youssouf Mouliom Mfopit, Judith Sophie Engel, Rolf Nimzyk, Andrea Schaffrath, Gloria Dada Chechet, Petra Berger, Mahamat Alhadj Moussa Ibrahim, Daniel Mbunkah Achukwi, Mohammed Mamman, Emmanuel Oluwadare Balogun, Mohammed Nasir Shuaibu, Junaidu Kabir, Barbara Reinhold-Hurek, Sørge Kelm

**Affiliations:** 1 Institute of Agricultural Research for Development (IRAD), Yaounde, Cameroon; 2 Department of Biochemistry, Ahmadu Bello University, Zaria, Nigeria; 3 Africa Centre of Excellence for Neglected Tropical Diseases and Forensic Biotechnology (ACENTDFB), Zaria, Nigeria; 4 MARUM-PANGAEA, University of Bremen, Bremen, Germany; 5 Centre for Biomolecular Interactions Bremen, Faculty of Biology and Chemistry, University of Bremen, Bremen, Germany; 6 Department of Public Health, University of N’Djamena, N’Djamena, Chad; 7 Immuno-Parasitology Section, TOZARD Research Laboratory, Bamenda, Cameroon; 8 Department of Veterinary Pharmacology and Toxicology, Ahmadu Bello University, Zaria, Nigeria; 9 Department of Veterinary Public Health and Preventive Medicine, Ahmadu Bello University, Zaria, Nigeria

**Keywords:** *Pseudomonas*, Whole-genome sequencing, Virulence factors, Comparative analysis, Antimicrobial resistance genes, Secondary metabolites

## Abstract

Bacteria of the genus *Pseudomonas* are Gram-negative and comprise of at least 107 diverse species. Certain members of the genus *Pseudomonas* emerged as major opportunistic human pathogens, and others have been used as biocontrol agents. Tsetse flies, the biological vectors of African trypanosomes, harbour diverse communities of microorganisms. Tsetse microbiota is of interest because of their possible effect on vector competence. The aim of this study was to explore genetic features of a novel *Pseudomonas* species strain CAM1A isolated from tsetse caught in Dodeo, Cameroon. The bacterium was isolated from tsetse fly, and the whole genome was sequenced on the Illumina Miseq platform. Sequences were assembled with SPAdes and annotated using RAST and PATRIC annotation pipelines. The draft genome, made of 61 contigs was found to be at least 5,848,000 bp long, with 5,339 coding sequences, a DNA G + C content of 64.3%, and at least 80 tRNA + rRNA genes. No plasmid was found. Taxonomic analysis based on whole-genome sequences (ANI and GGDC) and MLSA indicated that CAM1A represents a putative novel species closely related to *Pseudomonas soli*. Several antimicrobial resistance and efflux-mediated resistance genes were predicted, suggesting its ability to evade many antimicrobial therapies. Determination of secondary metabolites within the genome revealed seven different secondary metabolites distributed on 11 biosynthetic gene clusters. Various virulence factors were found, but the isolate was predicted to be a non-human pathogen.

**Supplementary Information:**

The online version contains supplementary material available at 10.1186/s12863-025-01398-z.

## Introduction

Tsetse flies are the only cyclical vectors of African trypanosomes, responsible for human African trypanosomiasis and animal African trypanosomiasis [1]. Tsetse flies harbour diverse communities of microorganisms, especially bacteria, mainly in the intestinal organs. During the long period of coevolution, microorganisms such as *Wiggleworthia glossinidia, Wolbachia* sp., *Sodalis glossinidius*, and *Spiroplasma* sp., developed a symbiotic interaction with the fly [2].

In addition to the endosymbionts, tsetse gut also harbours a diversity of environmentally acquired bacteria which vary considerably depending both on the tsetse species or subspecies and the geographic origin of the flies [3]. These bacterial genera include *Pseudomonas*, *Acinetobacter*, *Enterobacter*, *Enterococcus*, *Providencia*, *Sphingobacterium*, *Lactococcus*, *Staphylococcus*, *Chryseobacterium*, *Lactococcus*, *Arthrobacter*, *Bacillus*, *Bacillales*, Burkholderia, *Planococcaceae*, *Paenibacillus*, *Enterobacteriaceae*, *Pantoea*, and *Morganella* [4–6].

This microbial community influences several aspects of the tsetse’s physiology, including nutrition, fecundity and the immune system [7]. The interactions between tsetse flies and their microbiota are thought to play a critical role in shaping the flies’ capacity to transmit diseases. *Pseudomonas* species, in particular, could influence the vector competence of tsetse flies, making them an important focus for understanding disease dynamics [8, 9]. Previous studies have explored the microbiota of tsetse flies, identifying symbiotic relationships that may impact disease transmission. However, little research has focused on the role of *Pseudomonas* species in this context, which is critical for exploring new strategies for controlling trypanosomiasis.

Members of the genus *Pseudomonas* are gram-negative, rod-shaped Gammaproteobacteria which are highly diverse. The genus is phylogenetically divided into at least 18 subgroups comprising more than 100 species [10]. A phylogenomic analysis revealed four wider phylogenetic groups: *P. fluorescens, P. stutzeri, P. syringae, P. putida* [11].

The members of the genus are able to colonise various ecological niches [12]. Numerous new species have been described, which were isolated from different environments, including soil, water, sediments, air, animals, plants, fungi, algae, compost, and humans. Also, some recently described species are plant or animal pathogens [13].

Various methods are used in *Pseudomonas* taxonomy. These include phenotypic-based methods used not only for the characterisation of new *Pseudomonas* species but also for their identification. Several chemotaxonomic characteristics are also useful for bacterial characterisation [14]. Despite the usefulness of the phenotypic and chemotaxonomic features in *Pseudomonas* classification and identification, the most precise techniques for these purposes are the genomic-based approaches [13]. Regarding gene analysis, although the use of 16 S rRNA gene sequences remains the basis of bacterial classification, allowing the differentiation of the genus *Pseudomonas* from other bacterial genera, this gene is not discriminative at the species level in several phylogenetic groups of *Pseudomonas* [15], and MLSA (multilocus sequence analysis) shows a better resolution [1, 5].

*Pseudomonas* spp. are characterised by their low antibiotic susceptibility, attributable to the low permeability of the bacterial cellular envelopes and a concerted action of multidrug efflux pumps with chromosomally encoded antibiotic resistance genes [16]. *Pseudomonas aeruginosa* emerges as a major opportunistic human pathogen capable of causing a wide range of acute and chronic life-threatening infections, particularly in patients with compromised immune system.

Certain members of the genus *Pseudomonas* have been used as biological control agents, contributing to inhibition of crop-pathogenic microorganisms [17]. During root colonisation, the bacteria might induce systemic resistance in the host plant or outcompete other (pathogenic) soil microbes by producing siderophores that can give a competitive advantage in low-iron environments [18]. As bioremediation agents, **s**ome members of the genus are able to metabolise various chemical pollutants in the environment, into non/less toxic chemical entities [19]. Strains of *Pseudomonas fluorescens* were shown to be able to lyse *Trypanosoma cruzi* [20], causative agent of Chagas disease.

In addition to being ubiquitous in various environments, these ecological roles make *Pseudomonas* of special interest in environments where vector-host dynamics, such as in tsetse flies, are crucial for disease control.

Despite previous studies on tsetse microbiota, little is known about the potential role of newly isolated *Pseudomonas* species in trypanosomiasis control. This study seeks to bridge this gap by exploring the genetic features of a novel *Pseudomonas* species isolated from tsetse flies.

## Methodology

### Bacterial isolation

Tsetse flies used in this study were collected in Dodeo using biconical traps baited with acetone. The study area and fly trapping have been previously described [21, 22].

Life flies were dissected in a semi-sterile work environment, under normal atmospheric conditions in a self-constructed mobile field cabinet. Airflow was not controlled or filtered, but air turbulence was minimised. The inside of the cabinet, dissecting tools, and microscope slides were thoroughly cleaned with 70% ethanol and air-dried. Flies were dissected using microsurgery magnifying glasses on paper towels previously soaked with 70% ethanol. Flies were dipped in 70% ethanol for 10 s prior to dissection of the gut to sterilise the surface. The dry fly was then dissected in sterile PBS on a clean slide to remove the gut. It was homogenised in sterile 50 mM Tris buffer pH 9.0 using a motor-driven pestle (Kimble Kontes) in a microfuge tube. An aliquot (30 µL) of gut homogenate was added to 150 µL sterile Maramorosch and Mitsuhashi Insect medium (MMI) prepared according to [23] and inoculated with a sterile syringe into 2 mL tubes containing 1.9 mL of MMI-Agar (15 g/L) supplemented with 20% foetal calf serum (FCS).

Samples were incubated upside down in the field at room temperature before transport to the laboratory, where they were stored at 4 °C before further processing. Single colonies were isolated by repeated streaking on MMI-agar plates supplemented with 10% FCS. Plates were cultured at 30 °C under anaerobic conditions generated by Oxoid AnaeroGen sachets (Thermo Scientific, Dreieich, Germany). Pure bacterial cultures were isolated by repeated streaking and colony picking.

The genus of the bacterial isolates was initially established by 16 S rRNA sequencing. DNA was crudely extracted from bacterial isolates by bead-beating with 0.75–1 mm glass beads in 500 µL sterile 10 mM TE buffer pH 8.0 (10 mM Tris-Cl and 1 mM EDTA). Bead beating was repeated twice for 20 s. Supernatant was transferred in 1.5 mL microtubes and heated at 95 °C for 20 min to reduce potential spores within the samples. The bacterial 16 S rRNA gene was amplified with generic primers Bac8uf (5′-AGAGTTTGATNHTGGYTCAG-3′) and Univ1492r (5′-GGNTCCTTGTTACGACTT-3′) according to Grönemeyer et al. [24]. The approximate 1500 bp amplicons were then purified using the GeneJet Gel extraction kit (ThermoFisher) according to manufacturer instructions prior to sequencing by Seqlab, Göttingen, Germany. Obtained sequences were then subjected to BLAST (Basic Local Alignment Search Tool) searches at the NCBI (National Center for Biotechnology Information) data base to identify closest relatives.

### Purification of genomic DNA

Genomic DNA was isolated from the bacterial culture using a standard phenol-chloroform method with few modifications, as described previously [25]. The DNA dissolved in TE was quantified and checked for purity spectrophotometrically (NanoDrop^®^ Spectrophotometer ND-1000, ThermoFisher Scientific) and then stored at -20 °C. For integrity assessment, about 700 ng of DNA were run on 1% agarose.

### Genome sequencing

Genome sequencing was performed by AG Reinhold-Hurek, Dept. of Molecular Plant-Bacteria Interactions, University of Bremen, Germany. Briefly, extracted DNA was sheared by ultrasonication on Covaris M22 (conditions: 20% duty factor, 50 W, 45 s, 20 °C). DNA library preparation was performed using an Illumina TruSeq DNA PCR-free kit according to manufacturer instructions. The quality check was performed on an Agilent Bioanalyzer 2100 using a High Sensitivity DNA Kit, and DNA concentration was analysed by qPCR with the NEBNext Library Quant Kit (New England Biolabs). Sequencing was done on Illumina’s MiSeq using the MiSeq Reagent Kit v2 Nano with 150 bp paired-end. There were a total of 583,040 reads with a quality score of 89.9% >= Q30.

### Genome assembly and annotation

Reads were trimmed using Trimmomatic (Version: v.0.39), and sequences were assembled with SPAdes v3.15 [26]. All parameters were set to default except for the size of k-mers, which were manually chosen (-k 21,33,55,77,99,127). Gene prediction and annotation were performed using the Rapid Annotation System Technology (RAST) server available at http://rast.nmpdr.org [27]. The core of the RAST annotation engine, the SEED, houses subsystems (collections of functionally related protein families) and their derived FIGfams (protein families). The Bacterial and Viral Bioinformatics Resource Center (BV-BRC) information system, which used the circular viewer of the PATRIC (Pathosystems Resource Integration Center) server (https://www.bv-brc.org) [28], was also used. It circular viewer (circos-based) illustrated the circular representation of the genome.

### Identification and phylogeny

The entire 16 S rRNA gene sequence was extracted from the annotated genome and was submitted to BLAST (Basic Local Alignment Search Tool) analysis to determine the nearest phylogenetic neighbours against the NCBI GenBank database (http://www.ncbi.nlm.nih.gov/genbank/). The 16 S rRNA gene sequences of the closest neighbours (highest score) and some with average scores, were selected for phylogenetic analysis. *Escherichia coli* str. K12 substr. MG1655 (CP009685.1) was used as the outgroup organism. Phylogenetic analysis was conducted in MEGA X (Molecular Evolutionary Genetics Analysis) software [29]. The sequences were aligned using the ClustalW alignment tool [30] inbuilt with MEGA X. The phylogenetic tree was constructed based on the maximum likelihood method using the Hasegawa-Kishino-Yano model [31] and a discrete Gamma distribution. To assess the confidence or reliability of branches in the phylogenetic tree, 1000 bootstrap replicates were conducted, as this number of resampling provides a reliable estimate of support values while maintaining computational efficiency.

In addition, the bacterial species prediction tool (KmerFinder 3.2) using a fast K-mer algorithm available at https://cge.food.dtu.dk/services/KmerFinder/ [32, 33] was also used. The whole CAM1A assembled genome was submitted to the algorithm. The k-mer size used by KmerFinder for bacterial data is 16 bases.

### Whole genome comparative analysis

Whole-genome comparisons with other genomes were performed using different algorithms. Average nucleotide identity (ANI): ANI is a similarity index between a given pair of genomes, and the recommended cut-off point for species delineation is 95%. Whole genome sequences of the closely related neighbours (based on 16 S rRNA gene similarity) were downloaded from the Genbank and used to estimate the average nucleotide identity. The ANI and the Orthologous ANI (OrthoANI) values were calculated using the fIDBAC server [34] available at http://fbac.dmicrobe.cn/tools/ANI_calculator.

In addition, the genome sequence was uploaded to the Type (Strain) Genome Server (TYGS), available under https://tygs.dsmz.de, for identification, genome comparison, and phylogenetic inference [35]. Determination of closely related type strains was done by comparing our genome against all type strain genomes available in the TYGS database [36–38]. Genome-to-genome distance calculation (GGDC) was performed between genome pairs using the Genome BLAST Distance Phylogeny approach (GBDP) [39, 40].

### Multilocus sequence typing (MLST)

MLST was performed using a web-based method (MLST-2.0 server) available under www.cbs.dtu.dk/services/MLST [41]. The MLST genes were *acsA, argS, aroE, glnS, guaA, gyrB, ileS, mutL, nuoC, nuoD, ppsA, recA, rpoB, rpoD, and trpE* from *P. fluorescens*, *P. putida*, and *P. aeruginosa*.

### Multilocus sequence analysis (MLSA)

The following housekeeping (HK) genes were included in the MLSA assay: *atpD* (ATP synthase F1 beta subunit), *carA* (carbamoyl-phosphate synthase small chain), *gyrB* (DNA gyrase beta subunit), *ileS* (isoleucyl-tRNA synthetase), *recA* (recombinase A), *rpoB* (RNA polymerase beta subunit), and *rpoD* (RNA polymerase, sigma factor). These genes were selected based on the criteria that they are present as single copies in the genome and are homologous and ubiquitous in the studied taxa. The sequences of the seven housekeeping genes were extracted from the CAM1A complete genome.

Based on 16 S rRNA gene similarity and ANI values, 19 closely related *Pseudomonas* isolates were selected, and the amino acid (aa) sequences of the seven housekeeping genes were retrieved from the complete genomes of corresponding *Pseudomonas* isolates from the GenBank database. *Escherichia coli* str. K12 substr. MG1655 (CP009685.1) was used as the outgroup organism. Multiple sequence alignment of the aa was performed with CLUSTALW [30], and the sequences were trimmed manually for subsequent phylogenetic analyses. For phylogenetic analysis, the sequences of the seven protein-coding genes were concatenated into a single alignment (4,928 aa) using MEGA X [29] in the order *atpD* (460 aa), *carA* (378 aa), *gyrB* (807 aa), *ileS* (949 aa), *recA* (358 aa), *rpoB* (1359 aa), and *rpoD* (617 aa). The concatenated sequences of the seven loci were aligned using CLUSTALW [30], and a maximum likelihood tree was constructed in MEGA X [29] using the Le_Gascuel_2008 model [42] identified as optimal by the inbuilt model test module. A total of 700 bootstrap replicates were performed to evaluate the confidence or reliability of branches in the phylogenetic tree.

In addition, the CAM1A genome was submitted online to the Automated Multi-Locus Species Tree (autoMLST) sever available at https://automlst.ziemertlab.com. This server utilises Multi Locus Sequence Analysis (MLSA) to produce high-resolution species trees [43]. The autoMLST workflow starts by ANI estimation within the database. Estimated ANI values with reference genomes are found, which are used for organism selection. This set is then screened for single-copy genes present in every genome and prioritised based on MLSA criteria. Multiple sequence alignments are then obtained and trimmed. Final maximum-likelihood inference is calculated depending on the options and mode used [43].

### Antimicrobial resistance profiling and virulence factors

Predictions on antimicrobial resistance (AMR) phenotypes and virulence determinants of *Pseudomonas* sp. CAM1A were searched through the annotation data generated from RAST and also in the BV-BRC information system using the following tools: CARD and PATRIC (antibiotic resistance), VFDB, Victors and PATRIC_VF (virulence factors), and DrugBank and TTD (drug target).

To predict whether the organism is a human pathogen, PathogenFinder 1.1, available at http://cge.cbs.dtu.dk/services/PathogenFinder/ was used [44].

Predictive secondary metabolites were identified with AntiSMASH v.7.1 (antibiotics and Secondary Metabolite Analysis Shell) [45] available on https://antismash.secondarymetabolites.org/ and using strictness ‘relaxed’ parameters.

## Results

### Genome properties

The entire genome of the *Pseudomonas* sp. CAM1A was sequenced, and the sequencing coverage was 31.02X. The general molecular features of the genome are presented in Table 1.

**Table 1:** General molecular features

Size	5,848,000 bp
**Contigs**	61
**Plasmids**	0
**GC Content**	64.3
**N50**	467,642
**L50**	4
**Number of SEED Subsystems**	377
**Number of Coding Sequences**	5339
**Number of RNAs (**tRNA + rRNA)	> 80
**Protein Features** Hypothetical with functional assignments with EC number assignments with GO assignments with Pathway assignments with PATRIC genus-specific family (PLfam) assignments with PATRIC cross-genus family (PGfam) assignments	1,089 4,245 1,263 998 883 5,025 5,065

**Fig. 1:**
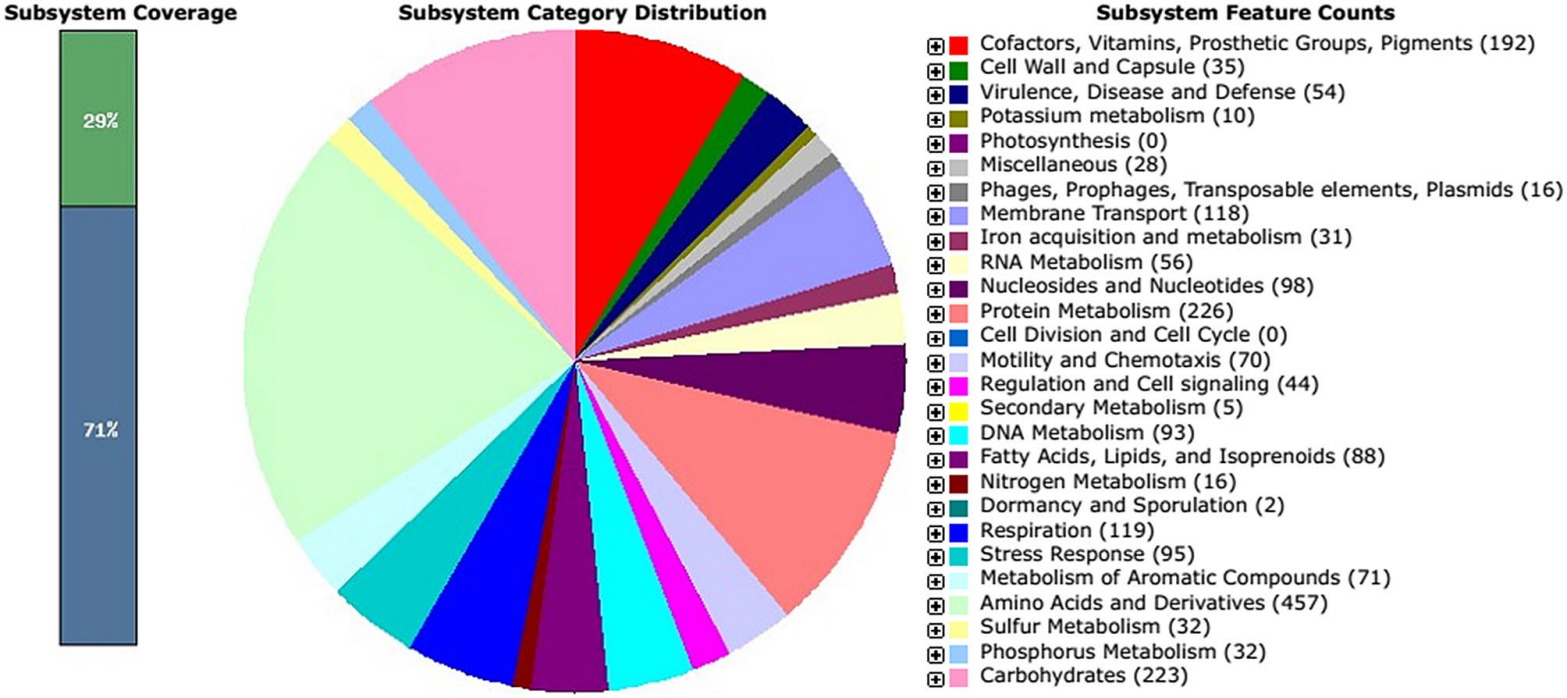
Overview of the subsystem categories of the annotated draft whole-genome of *Pseudomonas* sp. CAM1A from the RAST server. The pie chart shows the counts of genes related to each SEED-subsystem. The bar graph shows the subsystem coverage, this is the ratio of coding sequences annotated in the SEED subsystem (29%) and outside of the SEED subsystem (71%)

**Fig. 2:**
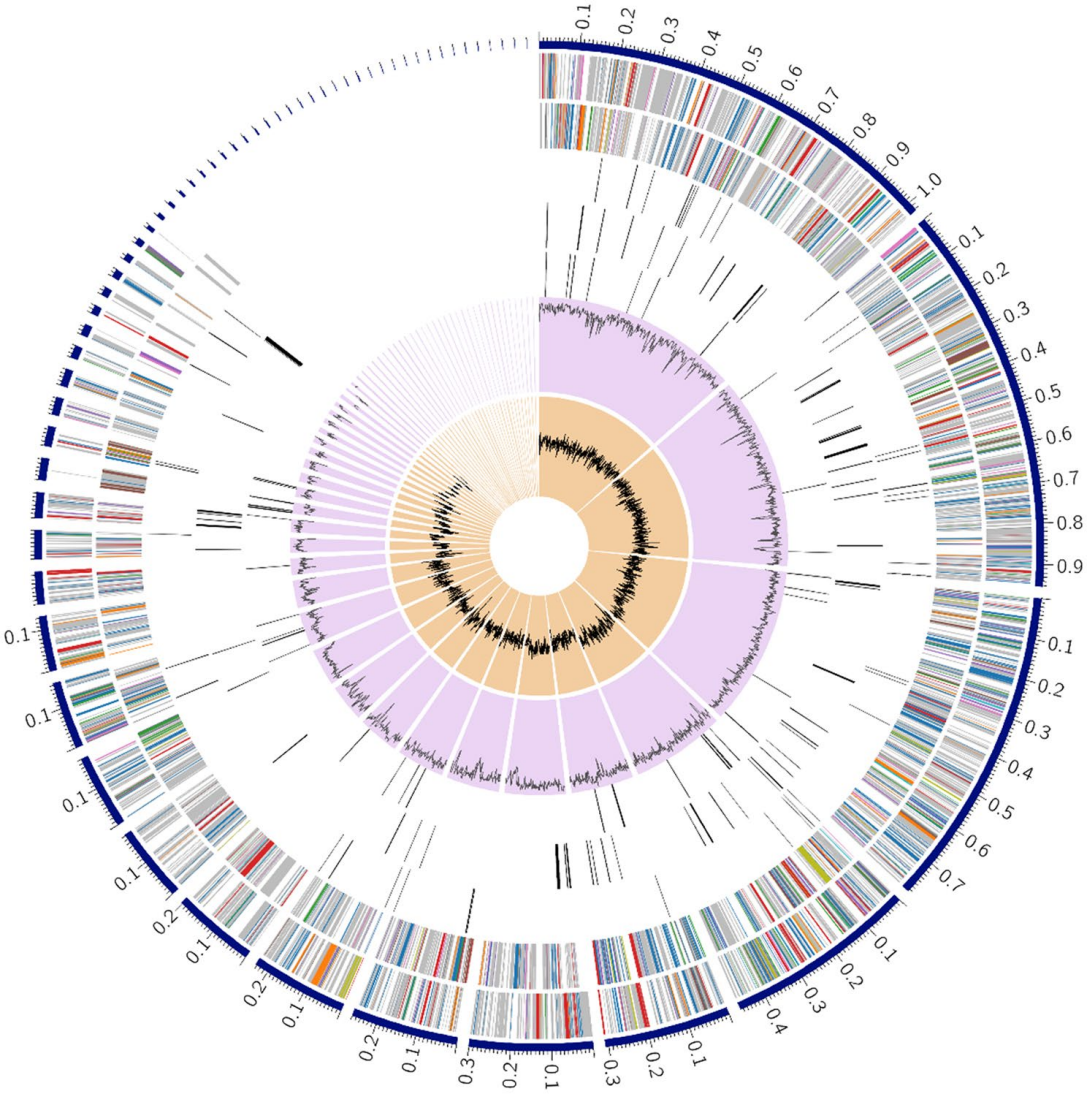
Circular graphical display of the distribution of the *Pseudomonas* sp. CAM1A genome annotation. This includes, from outer to inner rings, the contigs, CDS on the forward strand, CDS on the reverse strand, RNA genes, CDS with homology to known antimicrobial resistance genes, CDS with homology to know virulence factors, GC content, and GC skew. The colours of the CDS on the forward and reverse strands indicate the subsystem that these genes belong to (see PATRIC Subsystems Figure S1). The circular representation was provided by PATRIC annotation

The draft genome sequence of strain CAM1A consisted of 61 contigs and was at least 5,848,000 bases in length, with an average G + C content of 64.3%. Rapid genome annotation using the RAST annotation server described 5339 coding sequences and > 80 structural RNA genes (tRNA and rRNA). The coding sequences were classified into 377 SEED subsystems (Fig. 1), of which the most abundant were aa and derivatives (*n* = 457), protein metabolism (*n* = 226), carbohydrates (*n* = 223), cofactors-vitamins-pigments (*n* = 192), respiration (*n* = 119), membrane transport (*n* = 118), nucleosides and nucleotides (*n* = 98), stress response (*n* = 95), DNA metabolism (*n* = 93), fatty acids, lipids, and isoprenoids (*n* = 88), metabolism of aromatic compounds (*n* = 71), and virulence disease and defence (*n* = 54). Of the 5339 coding sequences identified, 29% were featured in SEED subsystems, while 71% were not. A circular graphical display of the distribution of the genome annotations is provided (Fig. 2).

### Taxonomy

The 16 S rRNA gene sequence subjected to a BLAST search at the NCBI database showed that it was closely related (99.87% sequence identity, 100% query cover, score 2841) to many other *Pseudomonas* isolates, including *Pseudomonas mosselii* (accession number CP024159), *Pseudomonas putida* (CP014343), *Pseudomonas* sp. CCOS 191 (LN847264) and *Pseudomonas* sp. B21-023 (CP087190). The 16 S rRNA phylogenetic tree showed our isolate to cluster with *Pseudomonas mosselii* (Figure S2). All the closest neighbours belonged to the *Pseudomonas putida* phylogenomic group. The 16 S rRNA gene sequence of *Pseudomonas* sp. CAM1A was deposited in the Genbank under accession number PQ650120.

For the identification of the isolate at the species level, kmerFinder was used. The closest neighbour in their database was found to be *Pseudomonas* sp. CCOS 191; but the query coverage (48.04%) and the template coverage (46.35%) were low (Table S1).

The ANI values obtained against other *Pseudomonas* spp. selected from the 16 S rRNA BLAST search are presented in Table 2. The analysis found the genome sequences of *Pseudomonas* sp. CCOS 191 (LN847264), *Pseudomonas* sp. RC3H12 (CP075595), *Pseudomonas* sp. B21-023 and *Pseudomonas* sp. B21-044 (CP087172) to have an ANI value slightly greater than the 95% cut-off point. Three *Pseudomonas soli* isolates were also at the border of the species delineation (94.9%). *Pseudomonas* sp. CCOS 191, considered as a novel species, was a close relative to *Pseudomonas mosselii* (ANI value) and also closely related to *Pseudomonas soli* based on MLSA [46]. *Pseudomonas* sp. B21-023 and the B21-044, another unclassified isolates, were also found to belong to the putida group [47].

**Table 2:** ANI values against related *Pseudomonas* spp

Name	Accession *N*°	Size (bp)	orthoANI	gANI
*Pseudomonas* sp. CCOS 191	LN847264	6,012,900	95.65	95.36
*Pseudomonas* sp. RC3H12	CP075595	5,760,142	95.14	94.9
*Pseudomonas* sp. B21-023	CP087190	5,874,598	95.1	94.95
*Pseudomonas* sp. B21-044	CP087172	5,727,781	95.09	94.99
*P. soli* strain SJ10	CP009365	6,247,860	94.9	94.65
*P. soli* strain NMI4264	CP128543	6,009,759	94.87	94.76
*P. soli* strain AP1	CP083803	5,583,152	94.85	94.65
*Pseudomonas* sp. 2hn	CP081016	5,901,931	92.18	91.78
*Pseudomonas* sp. LH21	CP144367	5,980,084	92.13	91.75
*P. mosselii* strain PH4	CP104107	5,807,827	91.93	91.54
*P. mosselii* strain PtA1	CP024159	5,742,165	91.88	91.59
*P. mosselii* strain BS011	CP023299	5,751,088	91.86	91.63
*P. mosselii* strain JP2-207	CP133092	5,702,443	91.79	91.5
*P. mosselii* strain 923	CP095556	5,755,630	91.73	91.52
*P. mosselii* strain DSM 17,497	CP081966	6,280,778	91.71	91.43
*P. muyukensis* strain COW39	CP077073	5,527,654	90.19	89.56
*P. xantholysinigenes* strain RW9S1A	CP077095	5,607,668	89.8	89.24
*P. entomophila* strain L48	CT573326	5,888,780	89.43	88.61
*P. xanthosomatis* strain COR54	CP077075	5,987,104	88.65	87.72
*P. fakonensis* strain COW40	CP077076	6,147,080	88.58	87.99
*P. asiatica* strain P1	CP084714	6,172,654	87.55	86.16
*P. putida* strain AKMP7	CP124529	5,764,016	87.52	86.15
*P. monteilii* SB3078	CP006978	6,000,087	87.47	86.42
*P. putida* strain PC2	CP011789	5,808,624	86.96	86.05
*P. parafulva* strain SE729	CP168118	5,747,255	86.89	85.74

The TYGS server was also used for species identification. Analysis is based on determination of the genome-to-genome distance (GGDC) between the query genome and all type strain genomes available in their database. Digital DNA-DNA hybridization (dDDH) values are used to determine the genetic distance between two organisms. DDH values below 70% indicate that the organisms belong to different species. The result from the TYGS server (Table S2) showed that the query genome did not belong to any species found in the TYGS database, suggesting our strain to be potentially a new species. The highest dDDH value (57.3%) was obtained with *Pseudomonas soli* LMG 27,941 although it is lower than the cut-off point of 70%.

### Multilocus sequence typing

The *Pseudomonas* sp. CAM1A genome was typed with three different MLST profiles available in the database (paeruginosa, pfluorescens and pputida). With the paeruginosa profile, no hit was found with six of the seven genes (*acsA, aroE, guaA, mutL, nuoD, ppsA, trpE*). Only *nuoD* matched with the input data (Table S3a). With the pfluorescens profile, using 7 genes (*glnS, gyrB, ileS, nuoD, recA, rpoB, rpoD*), no MLST loci were found in the input data (Table S3b). Using the pputida MLST profile with eight genes (*argS, gyrB, ileS, nuoC, ppsA, recA, rpoB, rpoD*), five matches were found (*argS, nuoC, ppsA, rpoB, rpoD*), indicating our isolate to be part of the *Pseudomonas putida* phylogenomic group (Table S3c).

### Multilocus sequence analysis (MLSA)

**Fig. 3:**
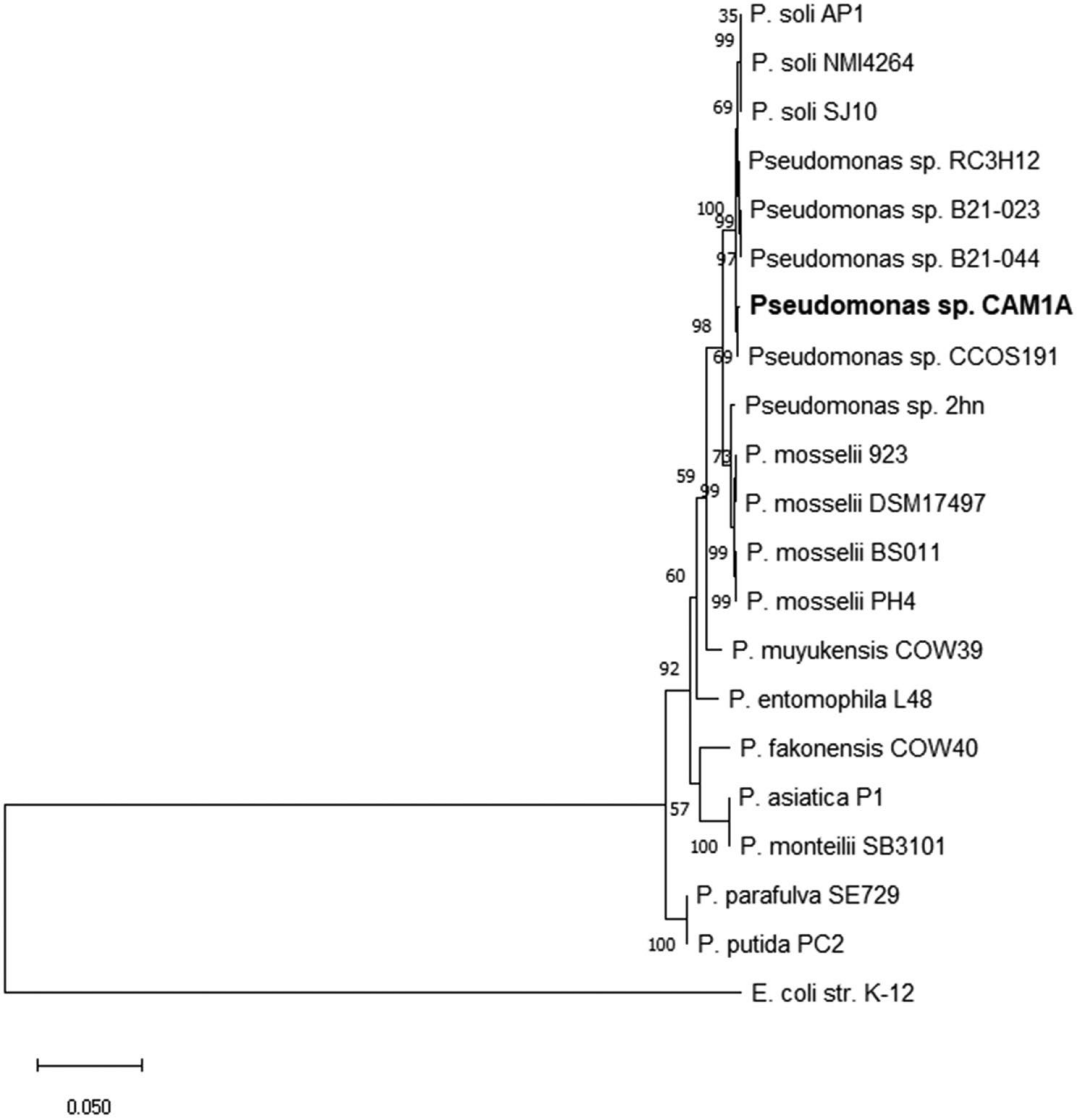
Multilocus sequence analysis tree. The analysis was based on aa sequence of seven concatenated HK genes (*atpD*, *carA*, *gyrB*, *ileS*, *recA*, *ropB*, and *ropD*). Our isolate is marked bold. The evolutionary history conducted in MEGA X [29] was inferred by using the Maximum Likelihood method and Le_Gascuel_2008 model [42]. A discrete Gamma distribution was used to model evolutionary rate differences among sites. Bootstrap analysis based on 1000 replications was used to estimate the confidence level of tree topologies. This analysis involved 21 aa sequences. There were a total of 4928 positions in the final dataset

A total of seven housekeeping genes (*atpD*, *carA*, *gyrB*, *ileS*, *recA*, *ropB*, and *ropD*) were analysed against 19 closely related *Pseudomonas* isolates. The individual genes for all the isolates were concatenated, which provided a unique sequence of 4,928 aa for each strain. Phylogenetic analysis of concatenated multilocus genes (Fig. 3) showed that our isolate clustered with 98% bootstrap support with *Pseudomonas* sp. CCO191 and forms a clade with 99% bootstrap support with the unclassified *Pseudomonas* strains (B21-023, B21-044, RC3H12) and *Pseudomonas soli* (SJ10, AP1 and NMI4264).

An online MLSA was also performed. The CAM1A genome was submitted to AutoMLST sever. A total of 84 genes (Table S4a) and 50 reference and query organisms were used in the final tree (Figure S3, Table S4b). The result showed that, the query sequence (PCAM1A) was closely related to *Pseudomonas* sp. CCOS191, followed by *Pseudomonas soli* LMG27941 then *Pseudomonas mosselii* SJ10 (later renamed as *Pseudomonas soli* SJ10 in 2018) [48].

### Carbohydrate-active enzymes

Carbohydrate-active enzymes, especially chitinases, are likely to play an important role in mediating interactions with the tsetse fly’s chitin-containing structures, such as the peritrophic matrix (PM). Based on the SEED viewer, we found in CAM1A genome, five enzymes linked to chitin and N-acetylglucosamine utilization: Glucosamine-6-phosphate deaminase [isomerizing], alternative (EC 3.5.99.6); chitin binding protein; Chitinase (EC 3.2.1.14); N-acetylglucosamine-6-phosphate deacetylase (EC 3.5.1.25) and a predicted transcriptional regulator of N-Acetylglucosamine utilization, GntR family.

### Antimicrobial resistance profile, virulence and pathogenicity

Genome analysis of CAM1A using SEED through the annotation data generated from RAST identified three major categories of genes associated with virulence, disease, and defense: (1) Tolerance mechanisms included genes conferring resistance to colicin E2. (2) Resistance to antibiotics and toxic compounds encompassed the MexC-MexD-OprJ multidrug efflux system, beta-lactamase, fluoroquinolone and fosfomycin resistance, as well as metal homeostasis and tolerance genes for copper, cobalt, zinc, and cadmium. (3) Invasion and intracellular survival genes included operons similar to *Mycobacterium* virulence clusters, implicated in ribosomal protein synthesis, DNA transcription, and quinolinate biosynthesis.

According to computational predictions in the BV-BRC information system, many genes annotated had homology to known transporters, virulence factors, drug targets, and antibiotic resistance genes (Table S5).

In addition, the predictions on AMR phenotypes was performed in PATRIC server, which used k-mer-based AMR genes detection method, assigned to each AMR gene broad mechanism of antibiotic resistance (Table S6).

The VFDB predicted 25 virulence factors in the CAM1A genome including numerous sequences encoding for flagella and Mycobacterium virulence operon. As for the pathogenicity, PathogenFinder predicted the *Pseudomonas* sp. CAM1A as non-human pathogen.

### Secondary metabolites

Determination of secondary metabolites within the PCAM1A genome revealed seven different secondary metabolites distributed on 11 biosynthetic gene clusters (Table 3). Identified secondary metabolites included hydrogen cyanide, ririwpeptide, kolossin, pseudomonine, tolaasin and 3-thiaglutamate.

**Table 3:** Identified secondary metabolite regions within *Pseudomonas* sp. CAM1A genome

Region	Type	Most similar known cluster	Sequence similarity
Region 1.2	hydrogen-cyanide	hydrogen cyanide	100%
Region 25.1	NRPS	ririwpeptide A/ ririwpeptide B/ ririwpeptide C	100%
Region 3.2	RiPP-like	3-thiaglutamate	80%
Region 35.1	NRPS	rhizomide A/rhizomide B/rhizomide C	100%
Region 39.1	NRPS	rhizomide A/rhizomide B/rhizomide C	100%
Region 40.1	NRPS	kolossin	100%
Region 41.1	NRPS	kolossin	100%
Region 44.1	NRPS	rhizomide A/rhizomide B/rhizomide C	100%
Region 47.1	NRPS-like	rhizomide A/rhizomide B/rhizomide C	100%
Region 9.1	NRPS	tolaasin I/tolaasin F	70%
Region 9.2	NRP-metallophore, NRPS	pseudomonine	100%

The prediction of the various compounds was performed using antiSMASH 7.0. using default setting of ‘relaxed’ stringency levels. Secondary metabolite clusters with 70% or higher similarity are listed

## Discussion

The interactions between tsetse flies and their microbiota are thought to play a critical role in shaping the flies’ capacity to transmit diseases [8, 9]. *Pseudomonas* sp. has been found in the gut of tsetse flies captured in different localities [4–6]. Many members of this genus displayed remarkable physiological and metabolic activity against different pathogens [17, 49]. They could influence the vector competence of tsetse flies, making them an important focus for understanding disease dynamics. This study aimed at isolating and exploring genetic features of a novel *Pseudomonas* species strain CAM1A. Whole genome sequencing has been used to provide solutions to problems in species identification, phylogeny, evolution, or pathogenicity. The entire genome of the isolated strain CAM1A was sequenced to assign it taxonomically, understand its molecular features, and obtain insights into its potential application for control of trypanosomiasis.

The strain was assigned to the genus *Pseudomonas* according to 16 S rRNA gene sequences. The draft genome sequence of *Pseudomonas* sp. still contained gaps but was at least 5.8 million base pairs (Mbp) in length, with a G + C content of 64.3%, at least 5339 coding sequences and 80 RNA genes. These values are similar to those obtained with many other *Pseudomonas* isolates where the size ranged from 5 Mbp to 7 Mbp, a G + C content of 62 to 66% and about 5000 to 7000 coding sequences [46, 47]. The number of subsystems differed from that of other isolates. The coding sequences were classified into 377 subsystems, which is lower than the 470 and 564 identified for *P. mosselii* Gil3 and *P. aeruginosa* ATCC 33,988 [50, 51] respectively. However, as the genome with 61 contigs is not complete, genes may still be missing. The genetic versatility of *Pseudomonas* spp. reflects the ubiquitous lifestyle of this bacterial species. The presence of genes related to carbohydrate and aa transport and metabolism is relevant for the fitness of environmental bacteria.

Based on the 16 S rRNA gene sequence, the analysis did not support a correct species affiliation of the strain. The isolate was closely related to *Pseudomonas mosselii, Pseudomonas soli and other non-classified Pseudomonas spp*. (CCOS191, B21-033, B21-044). This gene is not discriminative at the species level in several phylogenetic groups of *Pseudomonas* [15]. While 16 S classification has largely been a practical solution to taxonomic profiling, it can have the disadvantage of low resolution for closely related species. Therefore, analysis of the whole genome sequence provides a better resolution and thus a more robust assignment. The average nucleotide identity (ANI), genome-genome distance, and other pairwise comparisons are useful for discriminating *Pseudomonas* from other genera and for species-level identification. KmerFinder identified *Pseudomonas* sp. CCOS 191 as the closest neighbour.

ANI is a widely recognised measure of genomic relatedness, with an ANI ≥ 95% being the proposed cut-off point for species delineation [52]. In the present study, the ANI values were at the border of the species delineation. Indicating as closest species, *Pseudomonas* sp. CCOS 191. The closest classified species was *Pseudomonas soli*. Although classification via ANI emerged as a popular method to solve the taxonomy problems in bacteria, this similarity measure used alone still has difficulties resolving closely related strains. The MLSA methods are alternatives that can leverage several evolutionary markers. In this study, the results of the two MLSA analyses were similar and showed that our isolate was closely related to *Pseudomonas* sp. CCOS 191 and *Pseudomonas soli*.

Analysis based on whole-genome sequences (ANI and GGDC) and MLSA related our isolate to *Pseudomonas* sp. CCOS 191 and *Pseudomonas soli*. Accordingly, we may deduce that CAM1A represents a putative novel species closely related to *Pseudomonas soli*.

*Pseudomonas* sp. CCOS 191 was isolated from a water sample in Zürich, Switzerland, and was able to form large amounts of cyanide and dissolve phosphate minerals [46]. *Pseudomonas soli* SJ10 was isolated from wastewater of a nylon 6 production plant in Daegu, Republic of Korea. The strain was found to be efficient in the biodegradation of ε-caprolactam, sharing bioremediation characteristics with other *Pseudomonas* species [48]. However, our strain was isolated from the gut of a tsetse fly. In addition to the endosymbionts (*Wigglesworthia*, *Sodalis*, *Wolbachia*, and *Spiroplasma*), the tsetse gut also harbours a diversity of bacteria acquired from the environment. CAM1A is considered as environmentally acquired bacterium. This might result from feeding on an infected host or, more probably, from an infected water source, since the preferred habitat of tsetse is along rivers.

In previous studies using culture-dependent techniques, different bacteria genera have been isolated from tsetse gut, including *Pseudomonas*, *Acinetobacter*, *Enterobacter*, *Enterococcus*, *Providencia*, *Sphingobacterium*, *Lactococcus*, *Staphylococcus*, *Arthrobacter*, *Bacillus*, *Bacillales*, *Planococcaceae*, *Paenibacillus*, *Enterobacteriaceae*, *Pantoea*, *Morganella*, and *Providencia* [4–6]. However, little research has focused on their role in trypanosome transmission. *Pseudomonas* species produce a wide range of compounds with antimicrobial activity, and they have been used for biocontrol in plants [17]. They could also influence the vector competence of tsetse flies, making them an important focus for exploring new strategies for controlling trypanosomiasis.

The detection of genes involved in chitin and N-acetylglucosamine metabolism in CAM1A suggests a potential role in modulating the tsetse fly’s vector competence for trypanosomes. The presence of a chitinase and chitin-binding protein indicates the capacity to interact with or degrade chitinous structures such as the peritrophic matrix (PM), a key barrier influencing trypanosome establishment in the fly midgut [53]. Chitinolytic activity could compromise PM integrity, thereby facilitating parasite penetration, while chitin degradation products may further affect microbial or host metabolic signaling [54]. The associated GntR-family regulator implies environmental control of this pathway within the tsetse gut. These findings highlight a possible mechanism through which *Pseudomonas* sp. may influence trypanosome transmission. Targeting such bacteria or their chitin-degrading activities could provide a novel strategy to reduce tsetse vector competence and contribute to integrated approaches for trypanosomiasis control.

*Pseudomonas* spp. are known for their intrinsic resistance to many front-line antibiotics, due not only to their low outer membrane impermeability but also to antibiotic resistance genes and active efflux of antibiotics. This was confirmed following the genomic analysis of the resistance genes. The PATRIC and RAST servers predicted 76 genes linked to the resistance to antibiotics and toxic compounds.

The SEED analysis of the CAM1A genome revealed genes associated with virulence, disease, and defence, highlighting adaptations to survive within a challenging host environment. Genes conferring tolerance to colicin E2 and multidrug efflux systems suggest this bacterium can withstand antimicrobial pressures, likely providing a competitive advantage within the tsetse microbiome [55, 56]. Metal resistance genes, including those for copper, cobalt, zinc, and cadmium, indicate resilience to oxidative and metal stress generated during blood digestion, supporting persistence in the midgut. Antibiotic resistance determinants, such as beta-lactamase and fluoroquinolone resistance, point to environmental adaptability and potential as a reservoir for antimicrobial resistance. Interestingly, operons resembling *Mycobacterium* virulence modules linked to ribosomal protein synthesis, DNA transcription, and quinolinate biosynthesis may reflect mechanisms for intracellular survival or stress adaptation rather than true pathogenicity. In mycobacteria these modules have been shown to function in stress adaptation and persistence (e.g., co-regulation of ribosome maturation and cell-wall synthesis) [57, 58]. In the context of a gut symbiont rather than a pathogen, it is plausible these modules support intracellular survival or adaptation to the midgut environment rather than classic virulence. Collectively, these features suggest that PCAM1A is a metabolically versatile, resilient bacterium capable of enduring host defences and microbial competition. Its presence may influence tsetse microbiome stability, host physiology, and possibly vector competence, highlighting the ecological and functional significance of opportunistic bacteria in insect–microbe interactions. It was shown that colonisation of the tsetse fly midgut with commensal *Kosakonia cowanii* Zambiae inhibits trypanosome infection establishment [59]. A number of bacteria, such as *Pseudomonas aeruginosa*, with other bacteria (*Serratia marcescens*, *Providencia rettgeri*, *Bacillus thuringiensis*, and *Bacillus cereus*) were shown to induce mortality in *Glossina morsitans morsitans* [60]. *Pseudomonas* sp. was found to be associated with the uninfected status of *Glossina palpalis palpalis* in Cameroon [9].

Several coding sequences putative for mobile genetic elements (phage-related proteins) are located within the CAM1A genome. The mobilome contributes to the transfer of genetic material among bacteria. These genetic elements are believed to serve as a reservoir of new genes for the organism as the need arises and subsequently represent the adaptation of strains to changing environmental conditions [61].

Secondary metabolites or specialised metabolites are low molecular mass compounds produced naturally from the secondary metabolism of bacteria. They often play important roles in mediating interactions between the organisms that produce them and their host or other microbes [62]. Various gene clusters coding for non-ribosomal peptide synthetases (NRPSs) were identified within the CAM1A.

One of the NRSPs was similar to tolaasin, known to be produced by *Pseudomonas tolaasii*. Tolaasin is a lipodepsipeptide toxin, the cause of bacterial brown blotch disease of edible mushrooms *Agaricus bisporus* [63]. Tolaasin was shown to be phytotoxic when infiltrated into leaves of *Nicotiana tabacum* and was shown to be active against a range of basidiomycetes and Gram-positive bacteria [63]. Its effective secretion could contribute to competitiveness against non-related microbes in the gut microbiome.

A NRP-metallophore similar to pseudomonine (100% sequence similarity) was identified. Pseudomonine is an isoxazolidone with siderophoric activity. Siderophores are high-affinity iron chelating molecules produced by microorganisms to accumulate iron. They are also known as essential virulence factors in Gram-positive and Gram-negative bacteria [64]. However, a variety of siderophore-producing bacteria colonise the rhizosphere, promote iron uptake by plants, and subsequently promote growth [65]. Also here, competition of our strain for iron in the gut microbiome may be enhanced if this factor is produced.

Another secondary metabolite predicted in the CAM1A genome is hydrogen cyanide (HCN). *Pseudomonas* species are widely recognised for their potential in suppression of soil-borne pathogens. Their biocontrol ability has been attributed to the secretion of hydrogen cyanide [66]. Hydrogen cyanide has been shown to protect *Pseudomonas aeruginosa* against sodium hypochlorite-induced oxidative stress [67].

The effective production of these specialised metabolites could influence the vector competence of the fly. An antitrypanosomal factor has been shown to be produced by strains of *Pseudomonas fluorescens* which have been reported to be able to lyse *Trypanosoma cruzi* in vitro [20]. Several mechanisms could be hypothesised for the modulation of trypanosome infection by midgut *Pseudomonas* including reduction of tsetse lifespan, the competition for limited resources with parasites or the production of antiparasitic molecules.

## Conclusion

The whole genome sequence provided comprehensive insights into the genetic features of *Pseudomonas* sp. strain CAM1A isolated from a tsetse fly. Taxonomic analysis indicated that the isolate is a novel species closely related to *Pseudomonas soli*. Several important features, including virulence factors, antibiotic resistance genes, and biosynthetic gene clusters, were explored. This analysis suggests an organism adapted to survive in hostile environments, resist host immune pressures, and interact dynamically with other microbiota. The identification of genes involved in chitin and N-acetylglucosamine metabolism may have important implications for the tsetse fly’s vector competence for trypanosomes. Further investigations deserve to be undertaken in order to establish whether they can modulate the tsetse fly vector competence, which will be useful for new control strategies against trypanosomiasis.

## Supplementary Information

Below is the link to the electronic supplementary material.

Supplementary Material 1: KmerFinder results for species identification

Supplementary Material 2: Pairwise comparisons in Type Strain Genome Server

Supplementary Material 3: Pseudomonas sp. CAM1A MLST results

Supplementary Material 4: MLSA results by autoMLST server

Supplementary Material 5: PATRIC subsystems. Overview of the subsystem categories of the Pseudomonas sp. CAM1A genome from PATRIC annotation.

Supplementary Material 6: 16S rRNA Phylogenic tree of Pseudomonas sp. CAM1A. The phylogenetic tree was constructed based on maximum likelihood method using the Hasegawa-Kishino-Yano model [31], and a discrete Gamma distribution. The tree with the highest log likelihood is shown. This analysis involved 27 nucleotide sequences (the query sequence marked with a star and 25 nearest phylogenetic neighbours downloaded from the NCBI GenBank). There were a total of 1542 positions in the final dataset. A total of 1000 bootstrap replicates were performed. Evolutionary analyses were conducted in MEGA X [29].

Supplementary Material 7: Maximum-likelihood MLSA tree. The tree was generated automatically with autoMLST server. A total of 84 genes were involved in the analysis and 50 reference organisms were used in the final tree. The query organism is marked blue.

Supplementary Material 8

## Data Availability

The datasets generated and analysed during this study are included in this publication and its supplementary information file. The 16S rRNA gene sequence of Pseudomonas sp. CAM1A was deposited in the Genbank under accession number PQ650120. The whole-genome sequence data presented in this study have been deposited in GenBank as genome assemblies under BioProject accession number PRJNA1131784 and BioSample accession SAMN42321958.

## Abbreviations

AMR: Antimicrobial resistance
ANI: Average nucleotide identity
BLAST: Basic Local Alignment Search Tool
BV-BRC: Bacterial and Viral Bioinformatics Resource Center
FCS: Foetal calf serum
MLSA: Multilocus sequence analysis
MLST: Multilocus sequence typing
MMI: Maramorosch and Mitsuhashi Insect medium
NCBI: National Center for Biotechnology Information
PATRIC: Pathosystems Resource Integration Center
PCAM1A: Pseudomonas sp. strain CAM1A
RAST: Rapid Annotation System Technology
