# Supplementary figures and images for: Complete genome sequence and genetic features of a novel Pseudomonas sp. isolate (CAM1A) from tsetse fly gut captured in Dodeo, Cameroon
Source: BMC Genom Data. 2025 Dec 13;27:11. doi: 10.1186/s12863-025-01398-z (PMC12822146; doi:10.1186/s12863-025-01398-z)

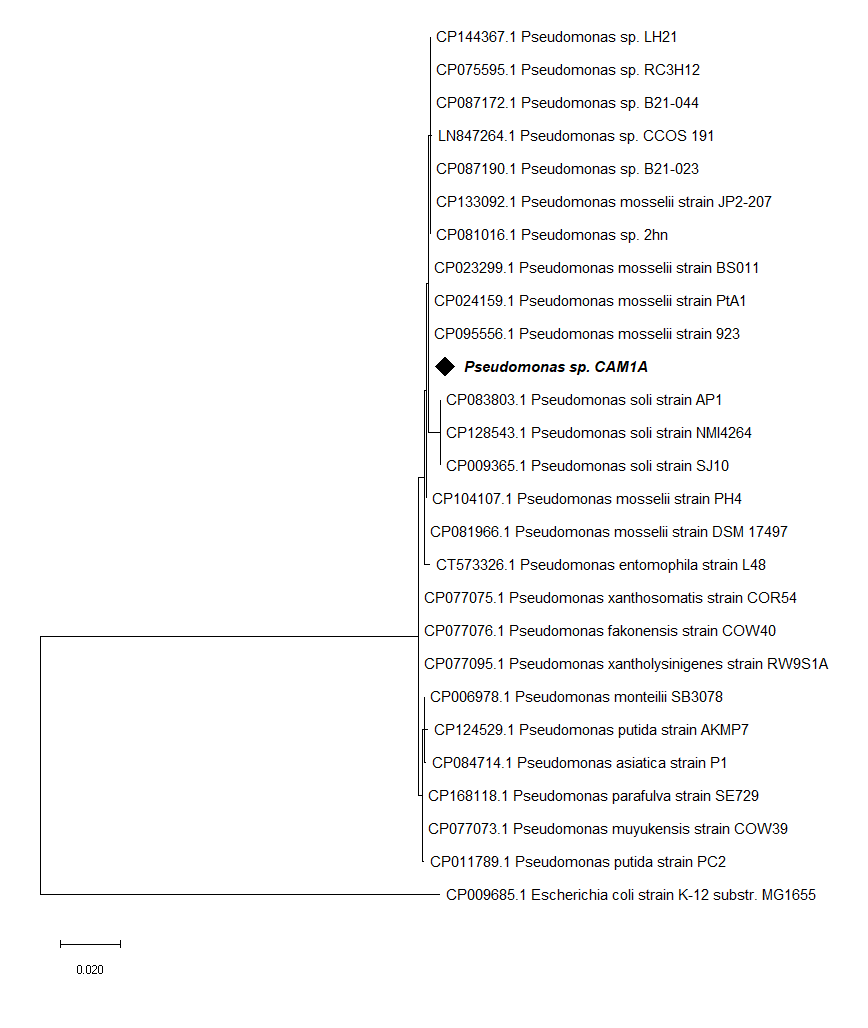

Supplement: Supplementary file 4 — Supplementary Material 4: MLSA results by autoMLST server [file 12863_2025_1398_MOESM4_ESM.png]

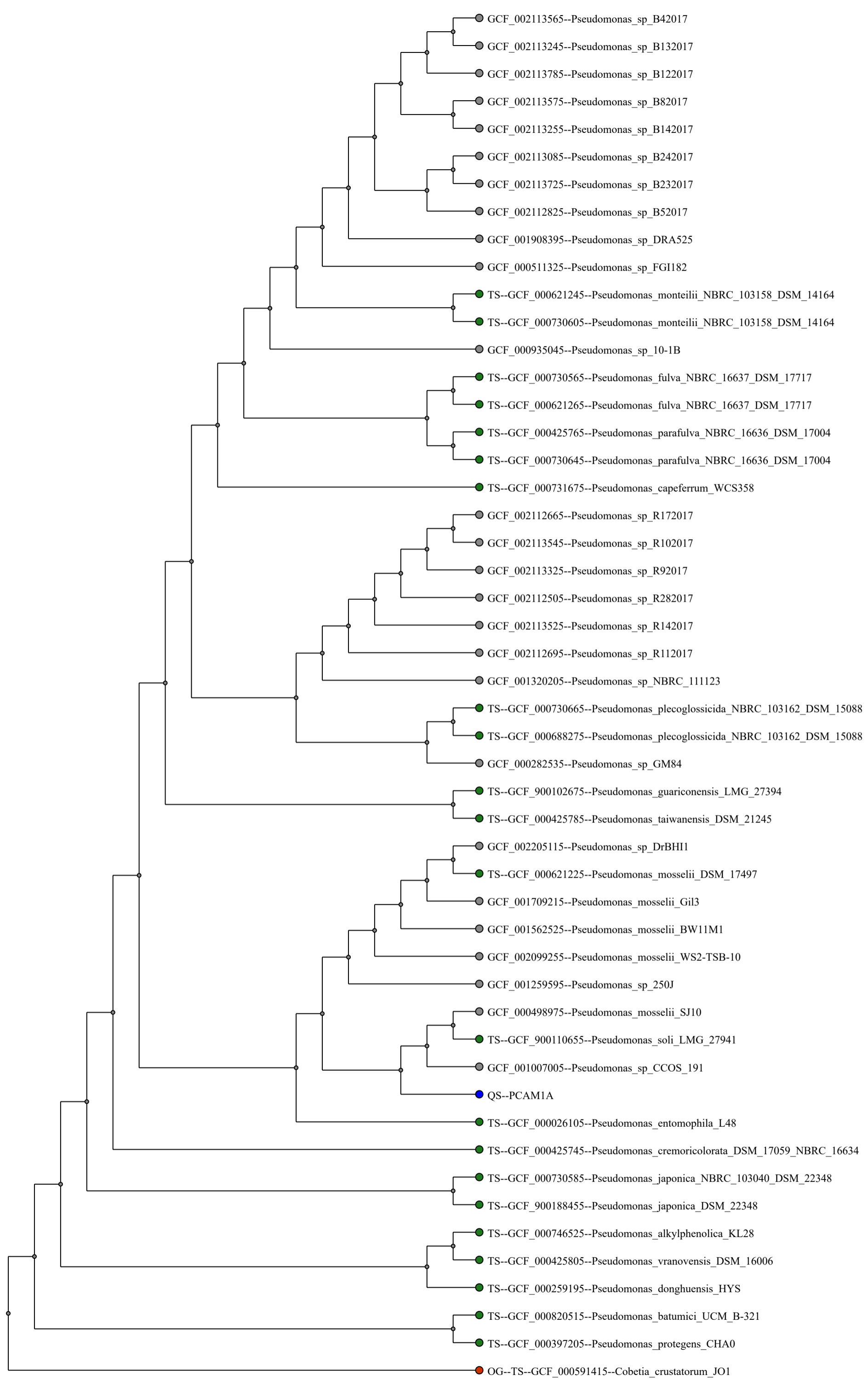

Supplement: Supplementary file 5 — Supplementary Material 5: PATRIC subsystems. Overview of the subsystem categories of the Pseudomonas sp. CAM1A genome from PATRIC annotation. [file 12863_2025_1398_MOESM5_ESM.pdf]

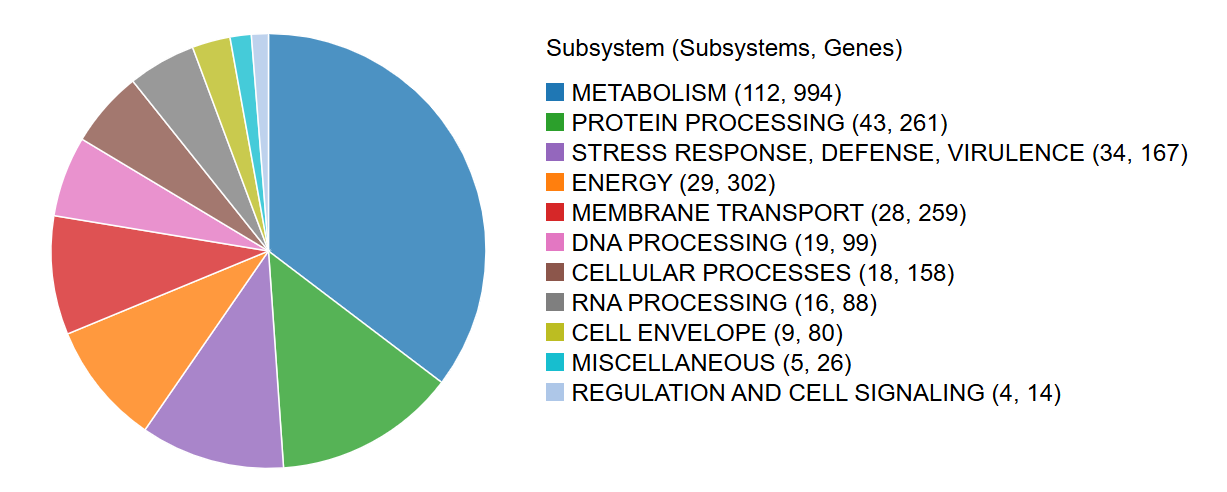

Supplement: Supplementary file 6 — Supplementary Material 6: 16S rRNA Phylogenic tree of Pseudomonas sp. CAM1A. The phylogenetic tree was constructed based on maximum likelihood method using the Hasegawa-Kishino-Yano model [31], and a discrete Gamma distribution. The tree with the highest log likelihood is shown. This analysis involved 27 nucleotide sequences (the query sequence marked with a star and 25 nearest phylogenetic neighbours downloaded from the NCBI GenBank). There were a total of 1542 positions in the final dataset. A total of 1000 bootstrap replicates were performed. Evolutionary analyses were conducted in MEGA X [29]. [file 12863_2025_1398_MOESM6_ESM.png]
