# Supplementary material for: Complete genome sequence and genetic features of a novel Pseudomonas sp. isolate (CAM1A) from tsetse fly gut captured in Dodeo, Cameroon
Source: BMC Genom Data. 2025 Dec 13;27:11. doi: 10.1186/s12863-025-01398-z (PMC12822146; doi:10.1186/s12863-025-01398-z)
Supplement: Supplementary file 8 — Supplementary Material 8 [file 12863_2025_1398_MOESM8_ESM.docx]

Table S5. Specialty genes and the specific source database where homology was found

|  | **Source** | **Genes** |
| --- | --- | --- |
| **Antibiotic Resistance** | CARD | 8 |
| **Antibiotic Resistance** | PATRIC | 79 |
| **Drug Target** | DrugBank | 31 |
| **Drug Target** | TTD | 7 |
| **Transporter** | TCDB | 70 |
| **Virulence Factor** | PATRIC_VF | 1 |
| **Virulence Factor** | VFDB | 25 |
| **Virulence Factor** | Victors | 22 |

Table S6. Antimicrobial resistance genes and corresponding mechanism.

| **AMR Mechanism** | **Genes** |
| --- | --- |
| **Antibiotic activation enzyme** | *KatG* |
| **Antibiotic inactivation enzyme** |  |
| **Antibiotic target in susceptible species** | *Alr, Ddl, dxr, EF-G, EF-Tu, folA, Dfr, folP, gyr*A*, gyr*B*, Iso-tRNA, kas*A*, Mur*A*, rho, rpo*B*, rpo*C*, S10p, S12p* |
| **Antibiotic target replacement protein** | *FabG, fabV, HtdX* |
| **Efflux pump conferring antibiotic resistance** | *EmrAB-TolC, MacA, MacB, MdtABC-OMF, MdtABC-TolC, MexAB-OprM, MexCD-OprJ, MexCD-OprJ* system, *MexEF-OprN, MexEF-OprN* system, *MexHI-OpmD, MexHI-OpmD* system, *MexJK-OprM/OpmH, MexVW-OprM, TolC/OpmH, TriABC-OpmH* |
| **Gene conferring resistance via absence** | *gidB* |
| **Protein altering cell wall charge conferring antibiotic resistance** | *GdpD, PgsA* |
| **Protein modulating permeability to antibiotic** | *OccD1/OprD, OccD2/OpdC, OccD3/OpdP, OccD4/OpdT, OccD6/OprQ, OccK10/OpdN, OccK5/OpdH, OccK8/OprE, OprB, OprD* family*, OprF* |
| **Regulator modulating expression of antibiotic resistance genes** | *OxyR* |
